# Functional guilds and drivers of diversity in seaweed-associated bacteria

**DOI:** 10.1093/femsmc/xtad023

**Published:** 2023-12-14

**Authors:** Tahsin Khan, Weizhi Song, Jadranka Nappi, Ezequiel M Marzinelli, Suhelen Egan, Torsten Thomas

**Affiliations:** Centre for Marine Science and Innovation & School of Biological, Earth and Environmental Sciences, The University of New South Wales, Sydney, NSW 2052, Australia; Centre for Marine Science and Innovation & School of Biological, Earth and Environmental Sciences, The University of New South Wales, Sydney, NSW 2052, Australia; Centre for Marine Science and Innovation & School of Biological, Earth and Environmental Sciences, The University of New South Wales, Sydney, NSW 2052, Australia; School of Life and Environmental Sciences, The University of Sydney, Sydney, NSW 2006, Australia; Centre for Marine Science and Innovation & School of Biological, Earth and Environmental Sciences, The University of New South Wales, Sydney, NSW 2052, Australia; Centre for Marine Science and Innovation & School of Biological, Earth and Environmental Sciences, The University of New South Wales, Sydney, NSW 2052, Australia

**Keywords:** symbiosis, guilds, taxonomy, seaweed, genomes, carbohydrate degradation

## Abstract

Comparisons of functional and taxonomic profiles from bacterial communities in different habitats have suggested the existence of functional guilds composed of taxonomically or phylogenetically distinct members. Such guild membership is, however, rarely defined and the factors that drive functional diversity in bacteria remain poorly understood. We used seaweed-associated bacteria as a model to shed light on these important aspects of community ecology. Using a large dataset of over 1300 metagenome-assembled genomes from 13 seaweed species we found substantial overlap in the functionality of bacteria coming from distinct taxa, thus supporting the existence of functional guilds. This functional equivalence between different taxa was particularly pronounced when only functions involved in carbohydrate degradation were considered. We further found that bacterial taxonomy is the dominant driver of functional differences between bacteria and that seaweed species or seaweed type (i.e. brown, red and green) had relatively stronger impacts on genome functionality for carbohydrate-degradation functions when compared to all other cellular functions. This study provides new insight into the factors underpinning the functional diversity of bacteria and contributes to our understanding how community function is generated from individual members.

## Introduction

Gene-centric metagenomic analyses of microbial communities from the same habitat often reveal high similarities in their functional gene profiles, despite phylogenetic or taxonomic variability. This has been observed, for example, for microbial communities associated with human guts (Qin et al. 2010), rivers (Frossard et al. 2012), soil (Mendes et al. 2015), seawater (Louca et al. 2016b), sponges (Fan et al. 2012), plants (Louca et al. 2016a), bioreactors (Wittebolle et al. 2008) and seaweeds (Burke et al. 2011, Roth-Schulze et al. 2018). Such functional equivalence on the community level can result from the stochastic assembly of individual communities from “functional guild” members that can be phylogenetically or taxonomically related to each other, or can come from distinct clades or taxa. (Simberloff and Dayan 1991). Multiple guild members may also exist in a single community, where they may act as “insurance” against the loss of a given guild function resulting from an organism's disappearance (Eisenhauer et al. 2012).

To reveal guild membership, the functionality of individual organisms needs to be assessed. Genome-centric functional analysis of metagenome-assembled genomes (MAGs) from different microbial communities, including those in anammox bioreactors (Ali et al. 2020), subseafloor aquifers (Tully et al. 2018), human guts (Forster et al. 2019) and sponges (Engelberts et al. 2020), have explored the existence of functional guilds. While useful, these studies have only considered one functional trait (e.g. nitrogen metabolism, sulfur cycling, carbon fixation, B-vitamin synthesis, or taurine metabolism) at a time to determined guild membership. However, an organism's ecological function needs to be assessed by the combination of all its traits or functions (Morris et al. 2020) and this can be achieved by assessing its total functional gene profile (Chauhan 2019).

Seaweeds (marine macroalgae) harbour complex communities of microorganisms on their surface, which appear to be essential for the hosts’ function, health and performance (Egan et al. 2013, Singh and Reddy 2016, Ghaderiardakani et al. 2020). Many studies have explored the taxonomic diversity of seaweed-associated bacteria in different environments and under various conditions (Saha et al. 2020b, Marzinelli et al. 2015, Morrissey et al. 2019, Capistrant-Fossa et al. 2021, Lemay et al. 2021, Ling et al. 2022, van der Loos et al. 2023, Zozaya-Valdés et al. 2017). Moreover, gene-centric metagenomic analyses of either different individuals of the same seaweed species (Burke et al. 2011), closely related species (Roth-Schulze et al. 2018) or distantly related species (Roth-Schulze et al. 2016) have revealed that individual hosts have large differences in the taxonomic composition of their bacterial communities, but similarity in their community functions. Therefore, seaweeds can be used as models to investigate guild membership in epibiotic bacterial communities.

The physiological traits of a seaweed host likely contribute to determining the functional features of its associated bacteria, including the polysaccharide-based cell wall constituents that may work as selective carbon sources for epibionts (Percival 1979), secondary metabolites capable of influencing colonization (Saha et al. 2020a, Kessler et al. 2018) and/or morphological features (Spoerner et al. 2012, Alsufyani et al. 2020, Lemay et al. 2020, Lemay et al. 2021). Apart from such host traits, bacterial taxonomy has also been found to determine the functional gene composition of individual bacterial species found in sponges (Robbins et al. 2021) and human guts (Forster et al. 2019). The actual drivers of the functional variation of the community members on seaweeds, however, still needs investigation.

To address these knowledge gaps, we generated an extensive data set of the taxonomic and functional diversity of bacteria associated with the three major seaweed groups (brown *Phaeophyceae*, green *Chlorophyta* and red *Rhodophyta*) and analysed a large collection of MAGs to examine functional guild membership and potential drivers of functional diversity. Specifically, if functional guilds are a feature of seaweed-associated microbial communities, we hypothesised that taxonomically distinct community members would have similar predicted functional profiles, and hence could be considered member of the same functional guild. We also hypothesised that if host traits are a driver of functional diversity, there would be an association between traits (such as carbohydrate metabolism) and functional variation between community members.

## Methods

### Metagenomic and genomic datasets

Publicly available raw metagenomic sequences for the microbial communities of the seaweeds *Amphiroa anceps* (Roth-Schulze et al. 2016), *Caulerpa filliformis* (Roth-Schulze et al. 2016), *Delisea pulchra* (Roth-Schulze et al. 2016, Zozaya-Valdés et al. 2017)*, Ecklonia radiata* (Roth-Schulze et al. 2016)*, Phyllospora comosa* (Roth-Schulze et al. 2016), *Ulva australis* (Roth-Schulze et al. 2016, Roth-Schulze et al. 2018b)*, Ulva ohnoi* (Roth-Schulze et al. 2018) *and Ulva rigida* (Roth-Schulze et al. 2018) were obtained through the Sequence Read Archive (SRA) database of the National Centre for Biotechnology Information (NCBI) (Agarwala et al. 2018) (Study accession number SRP065251, SRP087427, SRP094604 and SRP268394). Additional metagenomic sequences for the microbial communities of *Dilophus marginatus, Padina crassa, P. comosa, Sargassum linearifolium*, and *Sargassum vestitum* were obtained from the Earth Microbiome Project (EMP) (Shaffer et al. 2022). Previously generated MAGs from *E. radiata* obtained from (Song et al. 2021) were also included.

DNA from the surface microbiota of macroalgae, which were all freshly collected from the field, were previously obtained using the direct enzymatic lysis and extraction method (Burke et al. 2009) for *A. anceps, C. filliformis, D. pulchra, U. australis, U. ohnoi*, and *U. rigida* or from surface swabs followed by standard DNA extractions as described by Marzinelli et al. (2015) for *D. marginatus, E. radiata, P. crassa, P. comosa, S. linearifolium*, and *S. vestitum*. Metagenomic sequences from swabs of freshly collected *Hormosira banksii* were generated using the methods described in Nappi et al. (2022). Details about sample collection, DNA extraction method, sequence library preparation, sequencing platforms and accession number of the samples used in the study can be found in Table S1.

Publicly available genome sequences of cultured bacteria associated with marine macroalgae were obtained from the Genomes Online database (GOLD) (Mukherjee et al. 2019) and the NCBI database.

### Generation of metagenome-assembled genomes, taxonomic assignment and phylogenetic analysis

Metagenomic reads (Table S1) from multiple runs for a single sample were concatenated, then filtered for adapter sequences, and quality trimmed using Trimmomatic 0.38 (Bolger et al. 2014) with the setting of phred33 and SLIDINGWINDOW:4:20. Quality filtered reads were subsequently error corrected and assembled using metaSPAdes 3.13 (Bankevich et al. 2012) with default parameters. Quality-filtered reads were mapped onto the contigs using bowtie 2.3.4.2 (Langmead and Salzberg 2012) and coverage information for each contig was generated using samtools 1.9 (Li et al. 2009) and the *jgi_summarize_bam_contig_depth* (Kang et al. 2019) script.

Three different binning tools (MetaBAT 2.12.1 (Kang et al. 2019), MaxBin 2.2.3 (Wu et al. 2016) and MyCC 20170301 (Lin and Liao 2016)) were used with a minimum sequence cut-off of 2500 bp and otherwise default parameters followed with refinement using Binning_refiner v1.2 (Song and Thomas 2017). Completeness and contamination of all the genomes were assessed using CheckM 1.0.7 (Parks et al. 2015). Genomes were dereplicated within samples from the same seaweed species using dRep 2.3.2 (Olm et al. 2017) at 99% average nucleotide identity (ANI) setting. Only genomes of high (completeness > 90%, contamination < 5%) and medium (completeness ≥ 50%, contamination < 10%) quality were selected for further analysis following the standards described by Bowers et al. (2018).

The genome taxonomy database toolkit (GTDB-Tk) 0.3.2 (Chaumeil et al. 2019) was used to assign taxonomies to each MAG/genome based on the classification of genome taxonomy database (GTDB) (Parks et al. 2018). FastTree 2.1.10 (Price et al. 2010) was used to construct phylogenetic trees with the bacterial concatenated marker gene alignments provided by GTDB-Tk. The interactive tree of life (iTOL) v5 (Letunic and Bork 2019) was used to visualise trees using genome origin and their taxonomic classification.

### Functional annotation of MAGs

Prokka v1.14.5 (Seemann 2014) was used to predict protein sequences, which were annotated with the Cluster of Orthologous Groups (COG) (Galperin et al. 2021) (2020 release), the Kyoto Encyclopedia of Genes and Genomes (KEGG) (Kanehisa et al. 2019) Orthologies (KOs) (July 2018 release) and the Carbohydrate Active Enzymes (CAZy) (Lombard et al. 2014) (2020 release) databases. The COG and KEGG databases covered most of the known gene functions, whereas the CAZy database is specialised for carbohydrate metabolism. The Diamond v0.9.24 (Buchfink et al. 2014) tool was used with an E-value cut-off of 0.001 for similarity search against COG and KEGG. dbCAN 1.0 (Yin et al. 2012) was used with HMMER v3.2.1 (Mistry et al. 2013) with an E-value < 1e-18 and coverage >0.35 for annotation with the CAZy database. Annotations were performed with in-house python scripts (https://github.com/songweizhi/BioSAK).

### Data ordination and statistical analyses

Non-metric multidimensional scaling (NMDS) plots based on Bray-Curtis dissimilarity indices of the relative functional gene abundance per MAG were generated using the vegan v2.5–6 (Oksanen et al. 2015) and ggplot2 v3.3.0 (Wickham 2009) packages in R studio v1.2.5001 (Team 2014).

The R package mvabund v4.1.3 (Wang et al. 2012) was used to fit multivariate generalised linear models (GLMs) to test the effects of MAG taxonomy (i.e. phylum, class, order and family) or host type (i.e. seaweed group (i.e. brown, green, and red) or seaweed species) as fixed factors on the functional gene profiles of the MAGs. The tests were performed using the function “manyglm” for multivariate raw, non-transformed gene function count data, including the total count of annotated genes per genome as a model offset and a negative-binomial distribution, with adjusted p-values to account for multiple-testing and using 999 bootstraps. Residual plots, Q-Q plots and mean-variance plots were checked to ensure good model fits. Individual COGs, KEGGs (Level 4) or CAZy functions affected by each of the fixed factors was determined using a critical value of alpha=0.05. The significant effect of each model was determined by comparing fitted models with null models using likelihood ratio tests (LRTs).

## Results

### Taxonomic and phylogenetic diversity of MAGs from the seaweed microbiome

Using publicly available and newly generated metagenomic datasets for seaweed-associated microbiomes (Table S1), 4066 MAGs were generated for thirteen different marine macroalgal species. In addition, 659 previously generated MAGs from *Ecklonia radiata* were added to the analysis (Song et al. 2021). MAGs were dereplicated within seaweed species and this resulted in 389 MAGs of high quality (95.23 ± 2.69% completeness and 1.14 ± 1.06% contamination) and 913 of medium quality (69.95 ± 13.22% completeness and 2.82 ± 2.76% contamination) (Table 1). The average estimated MAG genome size was 3.84 ± 2.07 Mbp.

**Table 1.** Metrices for metagenome-assembled genomes (MAGs) used in this study.

Group	Seaweed species	High quality MAGs (Completion > 90%, Contamination < 5%)	Medium quality MAGs (Completion ≥ 50%, Contamination < 10%)	Total MAGs
Brown	*Dilophus marginatus*	2	4	6
	*Ecklonia radiata*	94	271	365
	*Hormosira banksii*	44	122	166
	*Padina crassa*	1	5	6
	*Phyllospora comosa*	28	117	145
	*Sargassum linearifolium*	1	2	3
	*Sargassum vestitum*	2	2	4
Green	*Caulerpa filiformis*	15	56	71
	*Ulva australis*	108	144	252
	*Ulva ohnoi*	6	10	16
	*Ulva rigida*	4	24	28
Red	*Amphiroa anceps*	7	31	38
	*Delisea pulchra*	89	123	212
Total		389	913	1312
Average completeness		95.23 ± 2.69%	69.95 ± 13.22%	
Average contamination		1.14 ± 1.06%	2.82 ± 2.76%	

**Figure 1.**
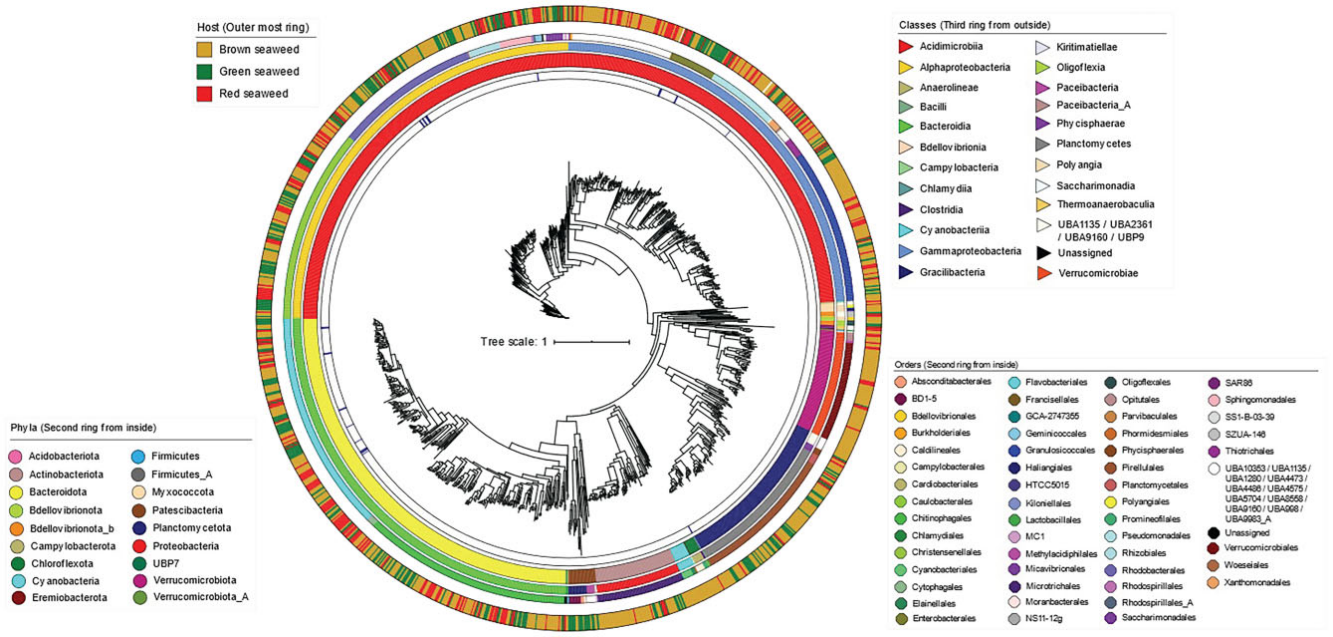
Phylogenetic tree of 1312 metagenomic assembled genomes (MAGs) and genomes of cultured bacteria associated with marine macroalgae. Blue strips in the inner ring mark genomes from cultured bacteria. Second, third and fourth ring from the inside represent phylum-, class-, and order-level taxonomy of MAGs with coloured explained in the figure panels. Outer ring is colour-coded by the major seaweed groups (brown, green and red).

Taxonomic and phylogenetic analysis of the 1312 MAGs revealed the presence of a diverse set of bacterial lineages (Fig. 1 and Fig. S1), which included a total of seventeen phyla dominated by the Pseudomonadota (previously Proteobacteria) (644 MAGs), Bacteriodota (323), Planctomycetota (129), Verrucomicrobiota (83) and Actinobacteriota (66) (together covering 94.89% of all MAGs) (Table S2). No archaeal MAG was recovered. The MAGs were further assigned to 25 classes, 64 orders and 88 families. Three MAGs (aa_83_Cluster.2, aa_83_metabat_bin.12 and er_BI_ER_110 816_Refined_6) could not be assigned to any known order. The number of MAGs not assigned to any described taxon increased further from the family level to the species level, where more than 98% of MAGs remained unassigned to a species (Fig. S2).

Only 19 genomes sequences for cultured bacteria from marine macroalgae were publicly available at the time of this study (Fig. S3). Bacteria from the phyla Pseudomonadota and Bacteroidota comprised most of these genomes and their diversity at lower taxonomic levels was limited. The genera *Aquimarina, Maribacter, Polaribacter, Alteromonas, Epibacterium, Phaeobacter* and *Ruegeria* were found in both the MAGs and the isolate genomes. In contrast, the genera *Arenibacter, Formosa, Maribacter, Polaribacter, Zobellia, Alkalibacterium, Ferrimonas, Kiloniella, Microbulbifer* and *Shewanella* were only found in the isolate genomes. No correlation was apparent between MAG phylogeny or taxonomy with host seaweed groups or species (Fig. 1 and S1).

### Functional gene profiles in the seaweed-associated bacteria

Seaweed-associated MAGs were annotated against three databases (COG, KEGG and CAZy) to describe their functional potential. Average annotations per detected open reading frame (ORF) for COG, KEGG and CAZy were 77.06% ± 9.17; 43.80% ± 9.50 and 2.17% ± 1.35, respectively, and 4305 functions were annotated against COG, 5943 for KEGG (Level 4) and 383 for the CAZy database.

**Figure 2.**
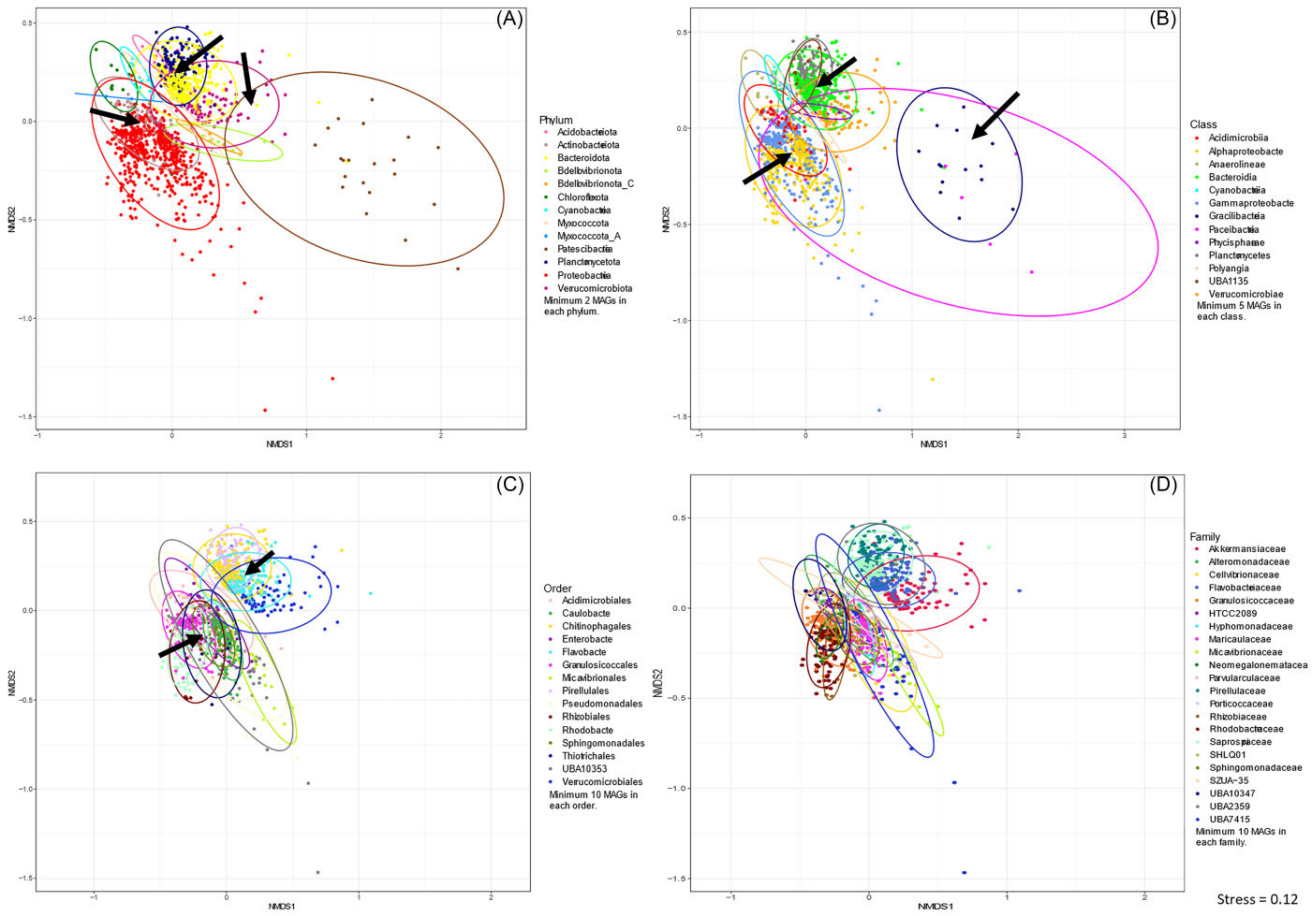
NMDS plots of functional gene profiles based on COG annotation of the MAGs. MAGs are coloured by their phylum (A), class (B), order (C) or family (D) level taxonomy. Phyla with a minimum of two, classes with a minimum of five, and orders and families with a minimum of 10 MAGs are plotted. Coloured ellipses depicting 95% confidence intervals around clusters of taxonomic groups. Arrows point to groups of MAGs where taxonomic clustering overlaps based on gene functions.

To observe the functional relatedness between MAGs, non-metric multidimensional scaling (NMDS) plots were generated based on the predicted gene profiles. Broad clustering of the MAGs based on their taxonomic grouping was observed for COG and KEGG (Level 4) annotations (Fig. 2 and S4, respectively). At the phylum level, several clusters overlapped forming groups where MAGs with different taxonomies share the same or very similar functional profiles (see arrows in Fig. 2). These overlaps included one group consisting of members from the phyla Acidobacteriota, Bacteriodota, Planctomycetota and Verrucomicrobiota, a second between Actinobacteriota and Pseudomonadota and a third dispersed group consisting of MAGs predominately belonging to the phylum Patescibacteria, but also containing members of the Bacteriodota and Verrucomicrobiota. At the class level, the Alphaproteobacteria and Gammaproteobacteria formed one group, as did the Bacteroidia, Physicphaerae, Planctomycetes, Verrucomicrobiae and UBA1135, and the Paceibacteria and Gracilibacteria clusters overlapped. At the order level, MAGs overlapped to form two main groups, one represented by the orders Caulobacterales, Enterobacterales, Granulococcales, Micavibrionales, Pseudomonadales, Rhizobiales, Rhodobacterales, Sphingomonadales, Thiotricales and UBA10353, and the other by Chitinophagales, Flavobacteriales, Pirellulales and Verrucomicrobioales. The family level plots followed similar patterns as the order level.

**Figure 3.**
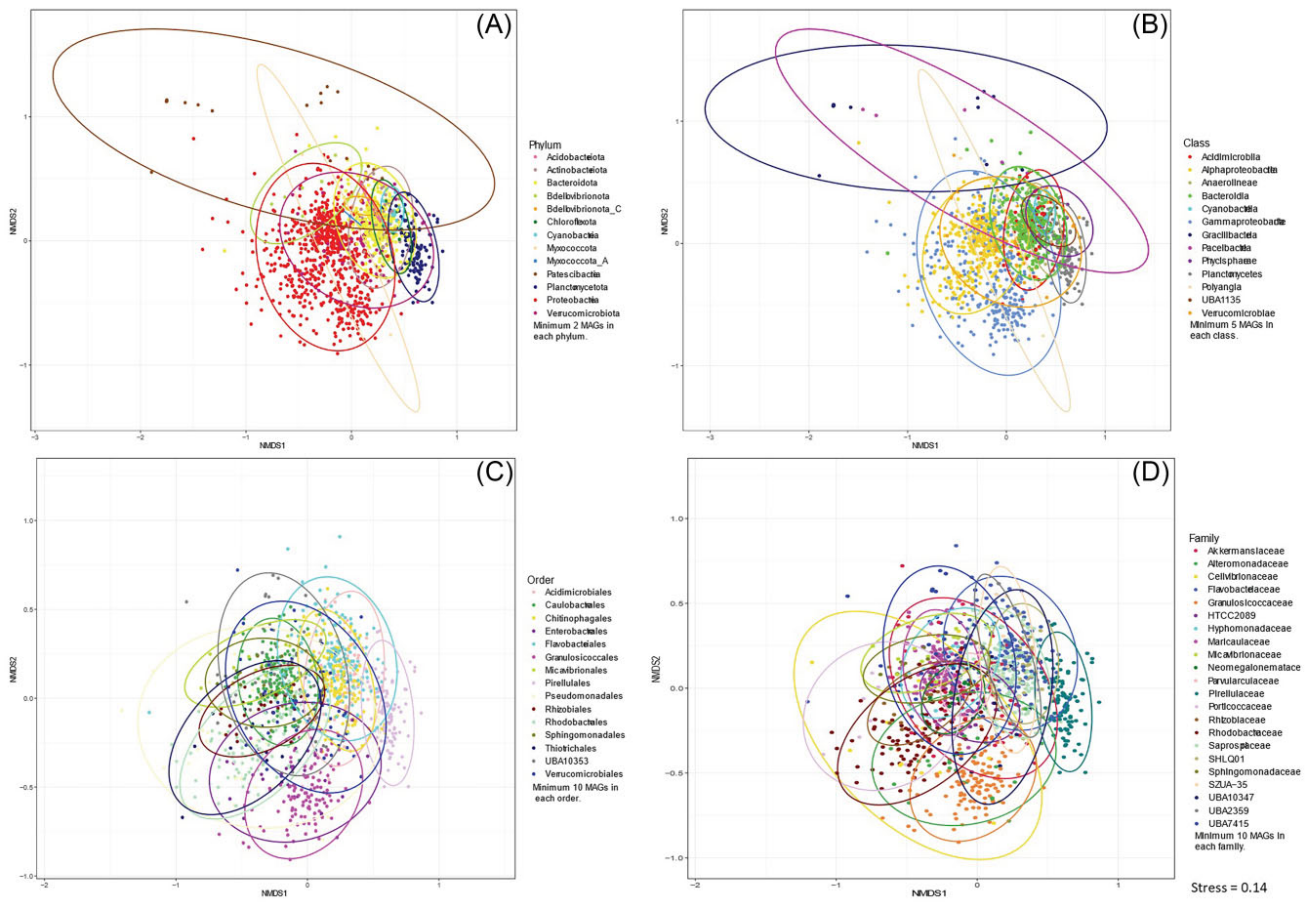
NMDS plots of functional gene profiles based on CAZy annotation of the MAGs. MAGs are coloured by their phylum (A), class (B), order (C) or family (D) level taxonomy. Phyla with a minimum of two, classes with a minimum of five, and orders and families with a minimum of 10 MAGs are plotted. Coloured ellipses depicting 95% confidence intervals around clusters of taxonomic groups.

**Figure 4.**
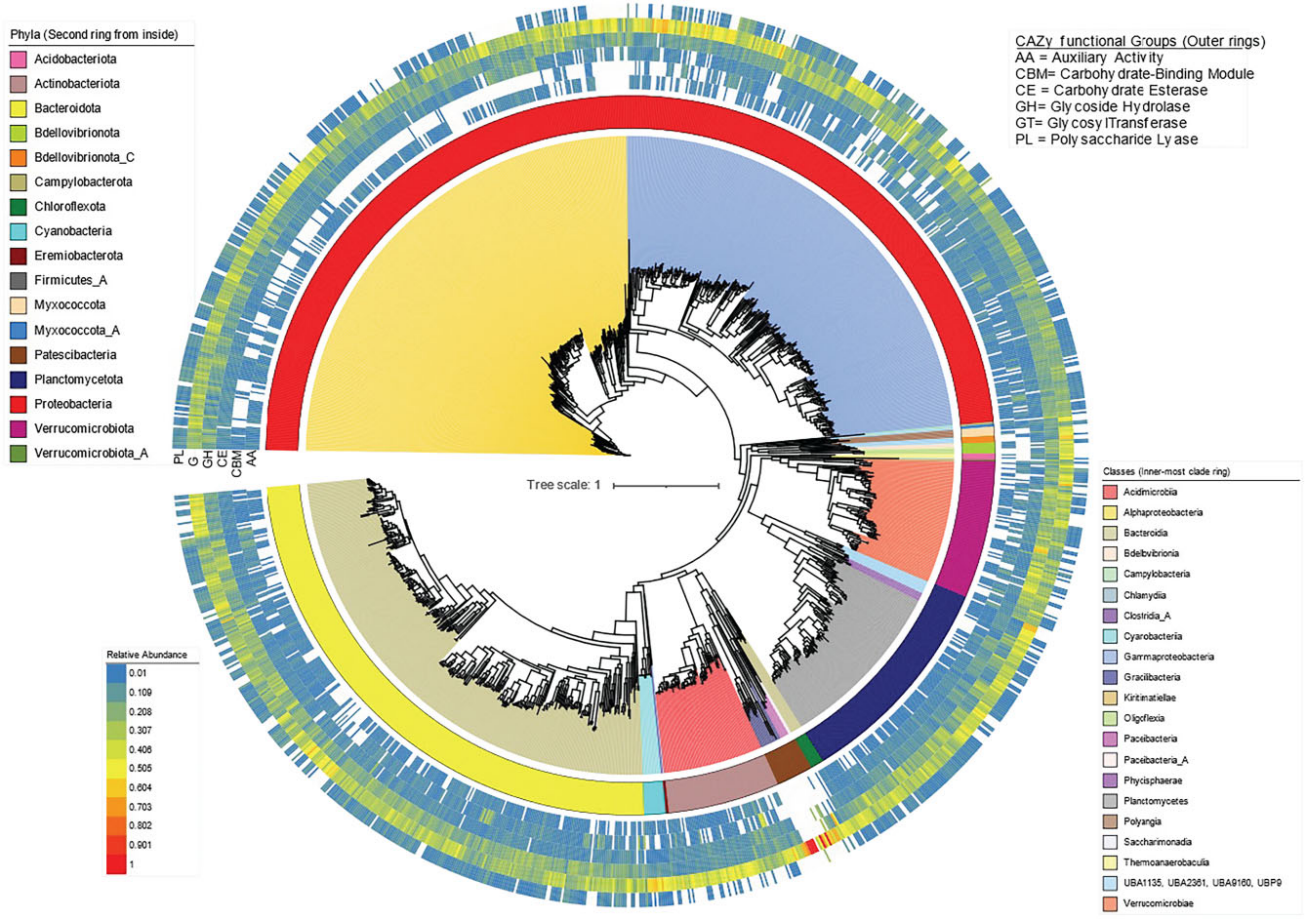
Phylogenetic distribution of CAZy functional groups across the MAGs. Inner segments show phylum-level taxonomy of MAGs, while inner ring shows class-level taxonomy. Colour coding is explained in panels. Second inner ring to outer ring represent the relative abundances of six CAZy functional groups. Relative abundances are coloured coded by heat map show at the bottom left. Labelling of the six rings on the left stands for functional CAZy groups listed at the top right. Only significant functions acquired from multivariate analysis using seaweed species as a fixed factor (n = 98) are shown. Functional diversity in seaweed-associated bacteria is characterised by taxonomy, carbohydrate metabolism and functional guilds.

For CAZy-based functional annotations, taxonomy-based clustering of MAGs was not as apparent as the patterns observed for COG and KEGG profiles. Instead, even at higher taxonomic levels the MAGs from different taxonomic groups substantially overlapped (Fig. 3). To further investigate the distribution of carbohydrate metabolism functions across MAG phylogeny, the relative abundances of CAZy gene functions (n = 98) that significantly differed between host types (i.e. brown, green and red seaweeds) were grouped into six broad functional categories (AA = Auxiliary Activity, CBM = Carbohydrate-Binding Module, CE = Carbohydrate Esterase, GH = Glycoside Hydrolase, GT = Glycosyl Transferase and PL = Polysaccharide Lyase). We found that CAZy functional categories were evenly distributed across the phylogenetic breadth of the MAGs (Fig. 4). This observation supports the notion that carbohydrate metabolic functions are weakly aligned with bacterial taxonomy in seaweed-associated bacteria and that different taxa could perform similar specialized functions in the context of carbohydrate utilization. However, some exceptions were observed, for example, MAGs from the phylum Patescibacteria were enriched for genes encoding glycosyl transferases (GT), but lacked other CAZymes. On the class level, the distribution of carbohydrate metabolic functions mostly followed the patterns of phylum-level though MAGs from the class Alphaproteobacteria had fewer CAZymes from the CBM group compared to other classes.

### Predictors of functional diversity in seaweed-associated bacteria

Multivariate analyses were used to assess and quantify the effect of seaweed group, seaweed species and bacterial taxonomy on the predicted functionality of seaweed-associated bacteria (Table 2). Bacterial taxonomy at different levels explained the variation of more than 3500 functions in both COG and KEGG annotations (or ∼ 63%–84% of all functions). Seaweed groups could explain the variation in ∼300 (∼6%—∼9%) COG and KEGG (Level 4) functions and seaweed species could explain the variation of ∼500 (∼12%) COG and ∼750 (∼13%) KEGG (Level 4) functions. Bacterial taxonomy could explain ∼50% of the carbohydrate metabolism functions, while seaweed groups and species explained ∼19% and ∼26% of functions, respectively. This shows that bacterial taxonomy is less of a driver relative to seaweed group or seaweed species when the functions for carbohydrate metabolism (CAZy) are considered compared to when all cellular functions (COG and/or KEGG) are taken into account.

**Table 2.** Multivariate analysis of factors influencing the seaweed-associated MAG functional profiles. Predictors were fitted as fixed factors to multivariate generalised linear models (GLMs) to explain functional gene profiles of MAGs. Likelihood ratio tests (LRTs) results and *P*-values are reported as well as the number of functions (and proportion of all functions in brackets), whose variation can be explained by the predictor. Analysis was done on different functional annotation systems.

Database		COG			KEGG (Level 4)			CAZy		
Annotated function no.		4305			5943			383		
Predictors		LRT	P-value	Significant function no. (%)	LRT	P-value	Significant function no. (%)	LRT	P-value	Significant function no. (%)
MAG taxonomy	Phylum	829 655	0.001	3440 (79.91%)	762 321	0.001	3785 (63.69%)	33 973	0.001	205 (53.52%)
	Class	1 009 775	0.001	3552 (82.51%)	941 029	0.001	3918 (65.93%)	41 618	0.001	215 (56.14%)
	Order	1 485 089	0.001	3633 (84.39%)	1 370 380	0.001	4039 (67.96%)	61 435	0.001	218 (56.92%)
	Family	1 589 168	0.001	3503 (81.37%)	1 427 729	0.001	3885 (65.37%)	66 713	0.001	220 (57.44%)
Host type	Seaweed Group	37 420	0.001	377 (8.76%)	40 993	0.001	339 (5.71%)	4336	0.001	71 (18.54%)
	Seaweed Species	125 469	0.001	533 (12.38%)	134 583	0.001	767 (12.91%)	10 042	0.001	98 (25.59%)

## Discussion

### Taxonomic diversity and phylogenetic breadth of seaweed-associated bacteria

The diversity of bacteria associated with different seaweed species has been previously explored mainly using 16S rRNA gene sequencing and gene-centric metagenomics (Saha et al. 2020b, Marzinelli et al. 2015, Roth-Schulze et al. 2018, Morrissey et al. 2019b, Tourneroche et al. 2020, Capistrant-Fossa et al. 2021, Lemay et al. 2021, Ling et al. 2022a, van der Loos et al. 2023, Zozaya-Valdés et al. 2017) and more recently using genome-centric analyses of the kelps *Macrocystis pyrifera* (Vollmers et al. 2017), *Ecklonia radiata* (Song et al. 2021) and *Nereocystis luetkeana* (Weigel et al. 2022). In this study, the majority (∼95%) of the 1312 MAGs from different seaweed species from all three major groups (i.e. brown, green, and red) could be assigned to one of five main phyla (Pseudomonadota, Bacteriodota, Planctomycetota, Verrucomicrobiota, or Actinobacteriota) consistent with previous studies. No MAGs were recovered for the Archaea, which have previously only once been reported for three seaweed species in low relative abundances (Trias et al. 2012). This indicates that members of the Archaea are not prominent in seaweed-associated microbial communities, and this contrasts with other marine holobionts, such as corals or sponges (Wegley et al. 2004, Robbins et al. 2021).

Some of the less dominant phyla observed in this study (i.e. Acidobacteriota, Chloroflexota, Cyanobacteria, and Firmicutes) have also been reported to be either less abundant (Saha et al. 2020b, Lachnit et al. 2011, Mancuso et al. 2016, Lemay et al. 2018, Morrissey et al. 2019, Parrot et al. 2019, Tourneroche et al. 2020) or rarely found on seaweeds (e.g. Bdellovibrionata (Liu et al. 2020) and Patescibacteria (Tourneroche et al. 2020)), although bloom or aquaculture conditions can impact the relative proportion of these taxa compared to field/wild samples (Califano et al. 2020). Interestingly, we also identified MAGs belonging to the novel candidate phylum Eremiobacterota, a group that consists of metabolic diverse individuals, including those rich in novel biosynthetic gene clusters. Ca. *Eremiobcterota* have been found in association with boreal mosses (Ward et al. 2019), Antarctic soils (Ji et al. 2021) and more recently seawater (Paoli et al. 2022), but to the best of our knowledge, not on seaweeds. Indeed, a significant degree of novel taxonomic diversity was found here as many of the MAGs remained unassigned to any described taxon, highlighting the value of using this approach to uncover novel bacterial biodiversity associated with seaweeds.

### Functional guilds in seaweed-associated bacteria

Some seaweed-associated bacteria from distinct taxonomic groups had functional gene profiles that closely resembled each other, yet were different from members of their own taxon, and this pattern was observed at various ranks (from phylum to family). This observation supports the notion of functional guilds consisting of taxonomically distinct bacteria in the seaweed microbiome. The lack of strict linkage between taxonomy and function was particularly striking when considering the carbohydrate metabolism functions, where CAZy functional groups were found evenly distributed among most phyla and classes. The wide distribution of GTs might be explained by their essential roles in the biosynthesis of cellular oligo- and polysaccharides, as well as protein glycosylation (Lairson et al. 2008) and hence is likely unrelated to any specific ecological function. However, GHs are often found in different marine bacteria capable of degrading various seaweed polysaccharides like fucoidan (Dong et al. 2017), ulvan (Reisky et al. 2019) and carrageenan (Ficko-Blean et al. 2017) and this might constitute a specific adaptation to a seaweed-associated lifestyle driven by carbohydrate degradation. The only exception here are the Patescibacteria, which are depleted of most CAZy functions, potentially due to their reduced genome size (Tian et al. 2020). Interestingly, MAGs from the class Alphaproteobacteria lacked genes responsible for the carbohydrate-binding module (CBM) compared to other classes. This was also noticed in marine particle-associated bacteria, where the relative abundance of genes encoding CBM functions in alphaproteobacterial isolates was lower than the gammaproteobacterial ones, and especially in the epipelagic regions (Zhao et al. 2020), where most seaweeds are found.

### Drivers of functionality of seaweed-associated bacteria

While bacterial taxonomy, seaweed group and seaweed species could, to different degrees, explain the distribution of gene functions in seaweed-associated bacteria, bacterial taxonomy was quantitatively the most prominent driver of functionality. This is similar to the patterns observed for COG functional categories in bacterial genomes from the GTDB database and a diverse set of environments, where 41.1% of functional variation could overall be explained by taxonomy (Royalty and Steen 2019). The variation explained by taxonomy furthermore also differed between functional categories, with, for example, inorganic ion, amino acid and coenzyme transporters being largely influenced by phylum-level taxonomy. However other functions might be uncoupled from or weakly influenced by taxonomy as has been seen, for example, for antibiotic resistance functions in aquatic environments (Fang et al. 2019).

Bacterial taxonomy explained here the variation of a lower proportion (<58%) of all specialized function related to carbohydrate metabolism (CAZy) than compared to all cellular functions (>79%). Instead, seaweed group and species explained relatively more of the variation for CAZy functions (19% and 26%, respectively). This partial decoupling of carbohydrate metabolism from taxonomy is consistent with observations made by Martiny et. al. (2013), who reported that the ability of carbon utilization of hundreds of bacterial isolates was phylogenetically highly dispersed. Similarly, the taxonomic rank of family was previously shown to be a poor predictor of the GH and PL CAZyme gene abundances in human-associated bacterial genomes (Cantarel et al. 2012). Together our data and these studies support the conclusion that bacterial taxonomy, especially at higher taxonomic ranks, can only weakly explain the distribution of carbohydrate metabolism in bacteria.

Carbohydrates are important mediators in the host-microbe interactions (Hooper et al. 2002) and their metabolism by microorganisms has been found to be influenced by hosts. For example, in the human gut, bacterial carbohydrate metabolism is mediated by the constituents of the diets (plant-based dietary fibres) and thus the host (Cronin et al. 2021). In the case of seaweeds, different groups (i.e. brown, green versus red) possess different polysaccharides (i.e. fucoidan, alginate, ulvan, agar, carrageenan, etc) as their cell wall constituents, whose availability and consumption could be considered a strong deterministic host trait influencing the assembly of epibionts (Egan et al. 2013). Previous studies using gene-centric metagenomics already revealed that the community-wide functional potential for degradation of specific polysaccharides was correlated with different types of seaweed hosts (Burke et al. 2011, Roth-Schulze et al. 2016), and here we show that this also determines the functionality of specific community members.

## Conclusion

In this study, the microbiome of 15 different seaweed species was found to harbour a vast range of novel bacterial taxa and future exploration of additional seaweed species will likely uncover even more taxonomic and functional diversity. Using a genome-centric approach, this study further revealed a high degree of functional similarity of taxonomically distinct bacteria, which supports the notion that functional guilds exist in the seaweed microbiome. However future experimental work will be required to define the exact membership of these guilds both in terms of taxonomy and function. Despite bacterial taxonomy explaining the variation of most gene functions across seaweed-associated bacteria, host seaweed type and seaweed species also make significant contributions to the functional diversity, particularly when specialised functions such as carbohydrate utilisation are considered. Our results provide an important framework for further research that will be required to determine the precise threshold of functional similarity required for guild membership and for which multiple organisms can be considered functionally redundant.

## Supplementary Material

xtad023_Supplemental_Files
