# Supplementary material for: Functional guilds and drivers of diversity in seaweed-associated bacteria
Source: FEMS Microbes. 2023 Dec 14;5:xtad023. doi: 10.1093/femsmc/xtad023 (PMC10781435; doi:10.1093/femsmc/xtad023)
Supplement: xtad023_Supplemental_Files [file xtad023_supplemental_files.zip › Khan_etal_supplementary_material_revised.docx]

**Supplementary Tables**

**Table S1:** Summary of sample names, accession number, sample collection, DNA extraction method, sequence library preparation, sequencing platform, read statistic and assembly statistics.

**Table S2:** Number of MAGs at each taxonomic level and the percentage of each taxon as a total of taxa at each level.

**Supplementary Figures**


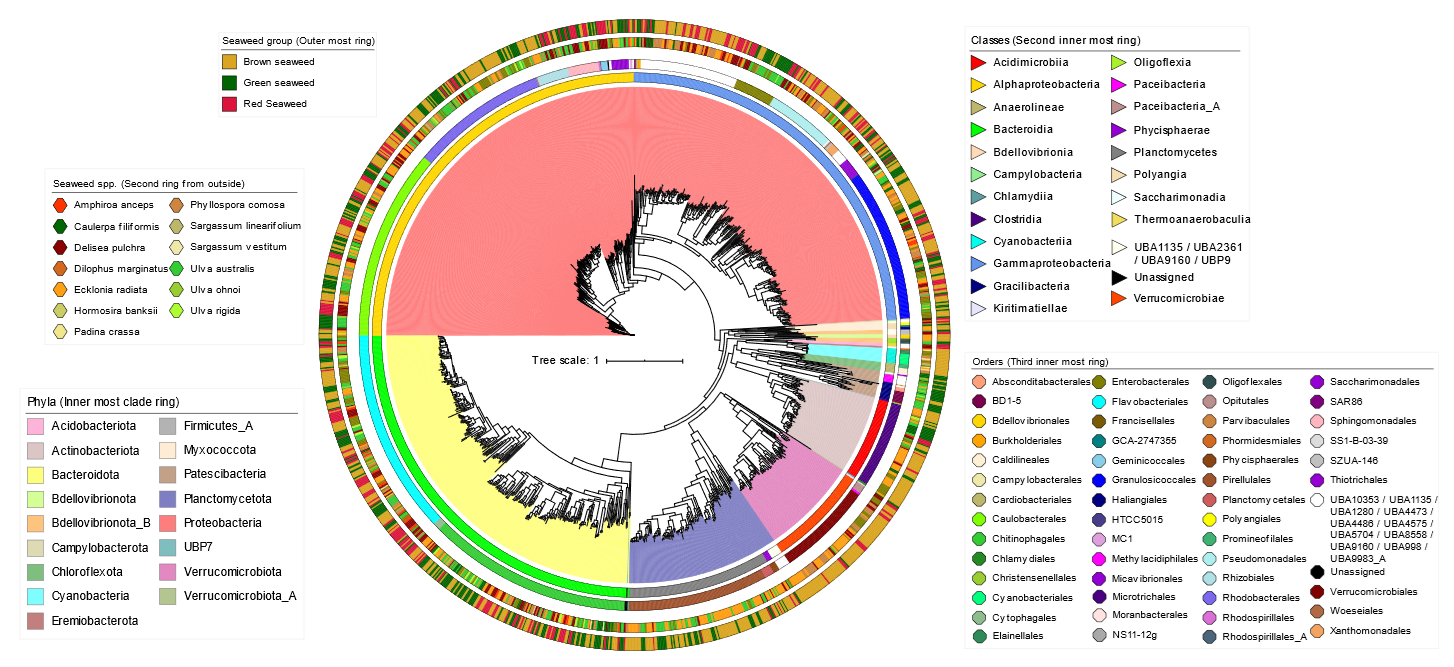


**Figure S1:** Phylogenetic tree of the 1312 metagenome-assembled genomes (MAGs) analysed in this study. Classification is based on the Genome Taxonomy Database (GTDB). Legends (from inner most ring: phyla, classes and orders, seaweed species and seaweed group) are colour coded alphabetically in descending order.

**
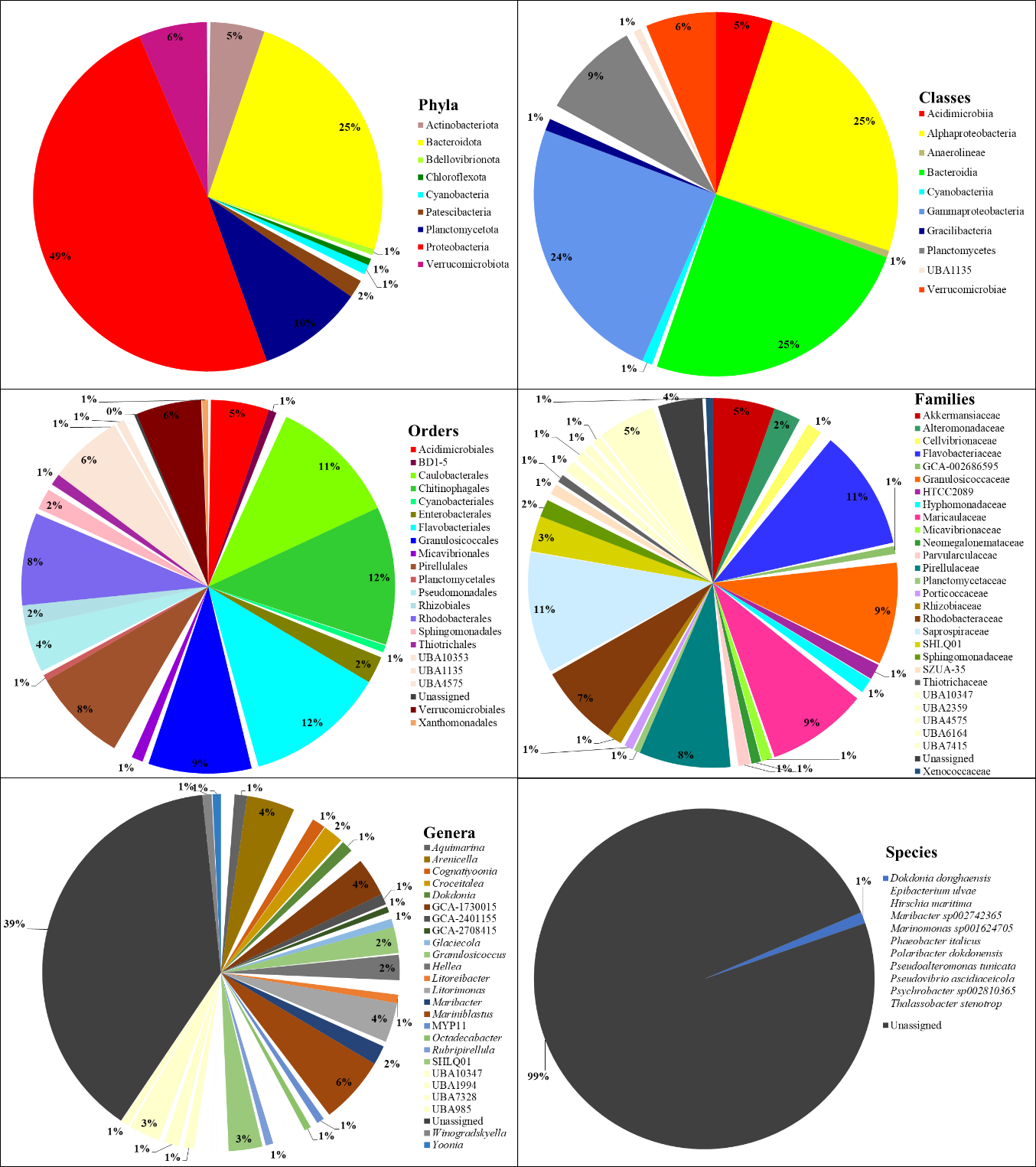
**

**Figure S2:** Pie charts showing MAG percentage at different taxonomic levels. Taxa with abundance of <1% are coloured white in the pie charts. The group of unassigned MAGs in each level are coloured in black.

**
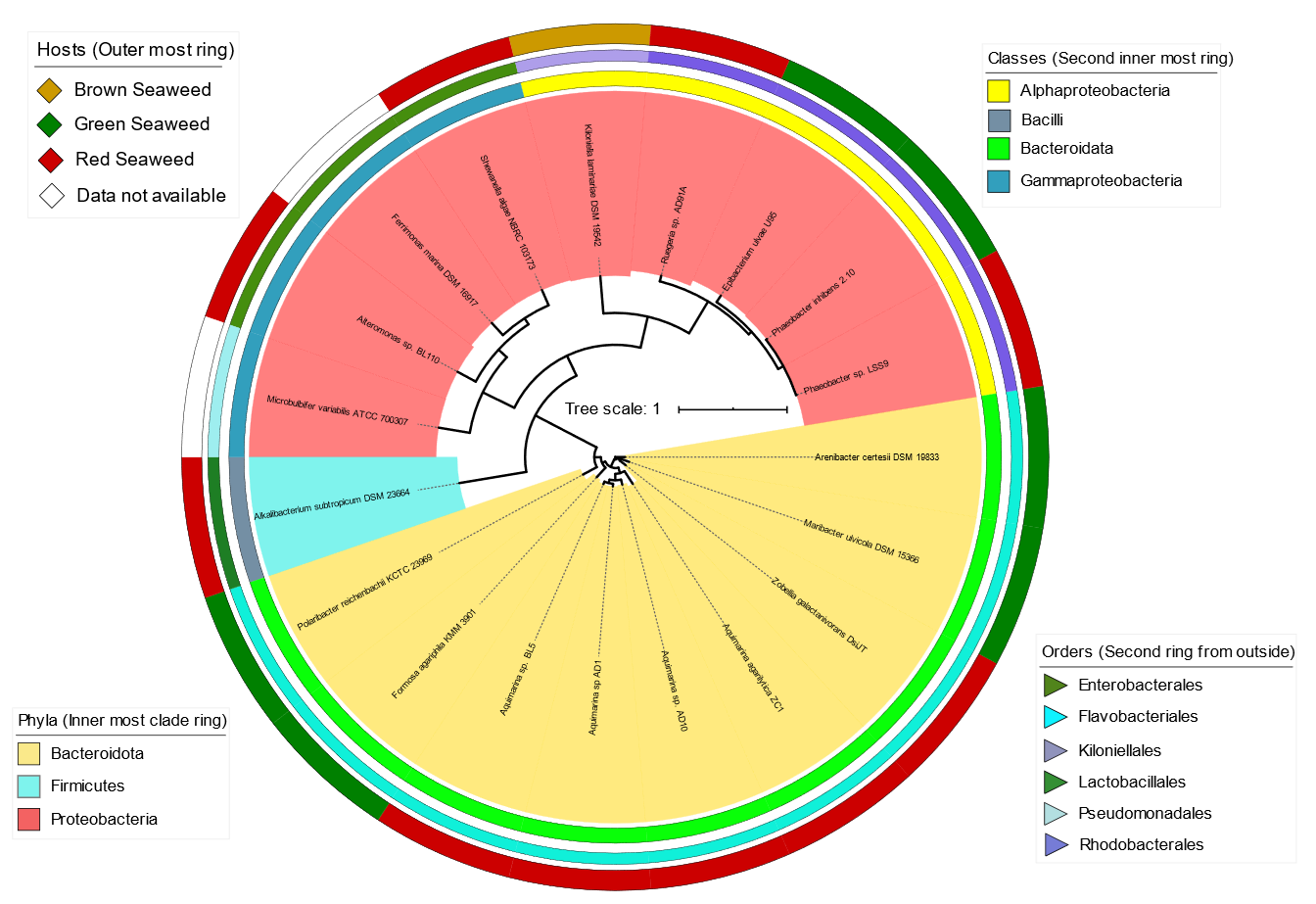
**

**Figure S3:** Phylogeny of the 19 publicly available cultured marine macroalgae-associated bacterial genomes analysed in this study. Classification is based on the Genome Taxonomy Database (GTDB). Legends (starting from inner most ring: host seaweed group, phyla, classes and orders) are colour coded alphabetically in descending order.

**
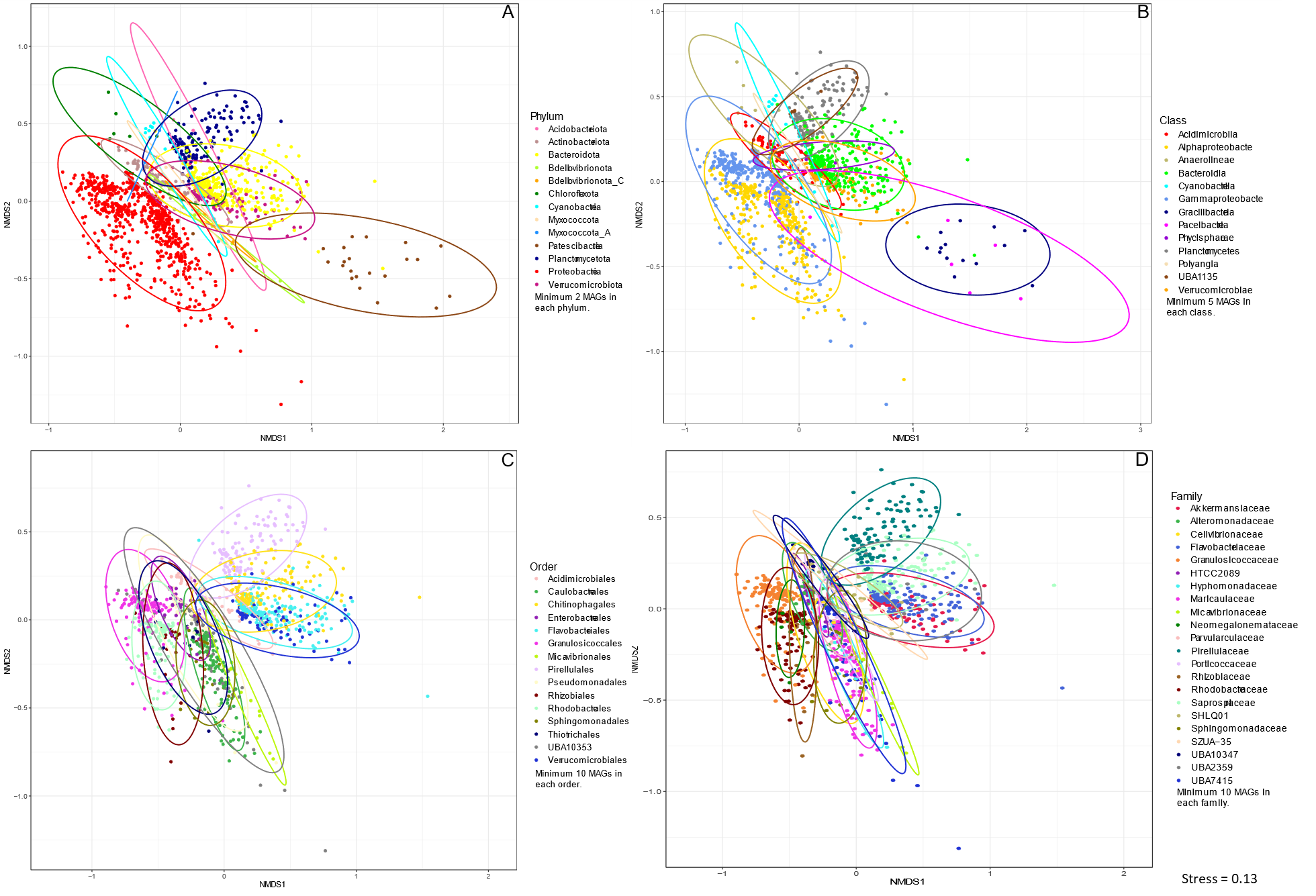
**

**Figure S4:** NMDS plots of functional gene profiles based on KEGG (Level 4) annotation of the MAGs. MAGs are coloured by their phylum (A), class (B), order (C) or family (D) level taxonomy. Phyla with a minimum of two, classes with a minimum of five, and orders and families with a minimum of 10 MAGs are plotted. Coloured ellipses depicting 95% confidence intervals around clusters of taxonomic groups.
